# Survival Outcomes in Hepatocellular Carcinoma Patients Undergoing TARE: A Comparative Analysis Before and After Single Admission Order–Map–Treat Protocol Implementation

**DOI:** 10.3390/cancers17243930

**Published:** 2025-12-09

**Authors:** Abdulmohsen Ahmed Alhussaini, Saleh AlShreadah, Mohamed Rajab Elzahrani, Abdulaziz AlTaweel, Mohammed AlAhmed, Omar Bashir, Shaker Al Shehri, Mohammad Arabi

**Affiliations:** 1 Division of Vascular and Interventional Radiology, Department of Medical Imaging, King Abdulaziz Medical City-Ministry of National Guard Health Affairs, Riyadh 11426, Saudi Arabia; alhussainiab@mngha.med.sa (A.A.A.); saleh.alshreadah@hotmail.com (S.A.); aaltaweel@kfshrc.edu.sa (A.A.); alahmedmo@mngha.med.sa (M.A.); bashirom@mngha.med.sa (O.B.); alshehrish1@mngha.med.sa (S.A.S.); 2 College of Medicine, King Saud Bin Abdulaziz University for Health Sciences, Riyadh 11481, Saudi Arabia; 3 King Abdullah International Medical Research Center, Riyadh 11481, Saudi Arabia; 4 King Faisal Specialist Hospital & Research Centre, Riyadh 11211, Saudi Arabia; elzahranimo@mngha.med.sa

**Keywords:** HCC, Y90, radioembolization, Order-Map-Treat, survival, Flexdose, single admission, mapping

## Abstract

**Background**: Hepatocellular carcinoma (HCC) continues to pose a significant global health burden, especially when diagnosed at a symptomatic or advanced stage. In such cases, prompt and well-coordinated treatment plays a key role in improving patient outcomes. This study explores the effect of introducing the Order–Map–Treat (OMT) protocol, designed to streamline clinical decision-making and minimize treatment delays, on the survival of HCC patients undergoing transarterial radioembolization (TARE). **Methods**: This retrospective cohort included 185 HCC patients (69.2% males), of which 88 (47.6%) underwent TARE before the implementation of the OMT system in 2021 (Group 1) and 97 (52.4%) afterwards (Group 2). The mean age of the entire cohort was 71 ± 12 years. A significantly larger number of patients treated before 2021 had an ECOG score of 0 (*p* < 0.001). Group 1 had significantly more multifocal disease, while group 2 had more unilobar involvement. More patients with PVTT3 and PVTT4 were treated after the implementation of the OMT protocol (*p* = 0.009). **Results**: The OMT protocol significantly reduced the median decision to treatment period (*p*-value ≤ 0.001) from 37 days to 15 days and mapping to the TARE period from 21 days to 1 day, shortening the total days needed for treatment by 32 days approximately. The median survival from TARE was 1.4 years (95% CI: 1.1 to 1.6) for the entire cohort. When stratified by treatment period, patients treated before OMT had a median survival of 1.5 years (95% CI: 1.2 to 1.9), while those treated after OMT implementation had a median survival of 1.2 years (95% CI: 0.9 to 1.6). The difference was not statistically significant (*p* = 0.415). **Conclusions**: While there were no significant survival benefits, the OMT protocol offers more efficient HCC management by minimizing delays in treatment, potentially improving patient experience and cost effectiveness.

## Simple Summary

The management of HCC requires a timely and multidisciplinary approach to achieve best clinical outcomes. This single institution study compared the survival outcomes prior to and following the implementation of the single admission Order-Map-Treat protocol. This protocol streamlines the management from decision to Y90 infusion and minimizes delays that could negatively impact clinical outcomes. While the study found no significant survival benefits, the OMT protocol proved more efficient by minimizing delays in treatment delivery. The median decision to treatment time was reduced to 15 days from 37 days, and the median mapping to treatment time was reduced to only 1 day from 21 days.

## 1. Introduction

HCC continues to pose a significant oncological challenge and remains a major concern in the field of medicine [1,2,3,4]. HCCs that are detected through routine surveillance often have a favorable prognosis, with a disease-free survival rate of over 50% at the five-year mark. On the other hand, HCCs that are detected after the onset of symptoms tend to have a poor prognosis, with a five-year survival rate of less than or equal to 10% [5].

One method of assessing the rate of HCC growth is by measuring the tumor volume doubling times (TVDTs), which involves comparing the tumor volumes obtained from consecutive imaging studies. A study conducted by An et al. aimed to measure the TVDTs of 175 HCC patients, and the results ranged from 11 to 851.2 days, with a median of 85.7 days [6]. Another systematic literature review conducted by Nathani et al. included twenty studies involving 1334 patients, and the pooled TVDTs ranged from 3.9 to 5.3 months [7]. These findings highlight the importance of timely intervention for patients with HCC [6,8,9,10].

Typically, a regular patient with HCC undergoes a series of steps before TARE, including discussion at a tumor board, admission, mapping, discharge, and readmission for the planned intervention [11,12,13]. However, this sequential process may lead to delays in patient management. Therefore, there is a need for a protocol that can expedite the management of HCC patients. One such protocol is the recently developed OMT protocol, which proposes that the patient be admitted, mapped, and treated in a single admission, thereby accelerating the overall management of the patient. The objective of this study is to evaluate the effectiveness of this newly adopted protocol by comparing the survival outcomes before and after its implementation. Assessing the protocol and its effectiveness will provide valuable insights to improve the efficiency of HCC management.

## 2. Patients and Methods

A retrospective cohort study was conducted to assess survival outcomes before and after the implementation of the OMT protocol.

All patients in the study were diagnosed with HCC at King Abdulaziz Medical City in Riyadh. These cases were discussed and approved by the local tumor board committee between the period of January 2018 to May 2023. The study data were collected and managed via REDCap electronic data capture tools hosted at the Medical Imaging Department of King Abdulaziz Medical City, Riyadh, Saudi Arabia [11,14,15]. Data were retrospectively retrieved from the electronic medical records and radiology information systems. Baseline clinical, biochemical, and imaging findings were retrieved. Portal vein tumoral thrombosis (PVTT) was classified according to Cheng’s classification [15]. The interval between MDT to TARE, mapping to TARE, and survival from first TARE were calculated.

The recently introduced OMT protocol was applied in December 2020 with the availability of the SIR-Spheres^®^ Y-90 resin microspheres FLEXdose Delivery Program (SIRTex, Woburn, MA, USA). In the OMT protocol, patients were admitted, underwent hepatic mapping with lung shunt study, and received treatment the following day within a single hospital stay. The Y90 dose is administered on day 2 pre-calibration to maximize the specific activity and reduce the potential embolic effect related to the number of microspheres. This streamlined approach aimed to expedite patient management and reduce the overall treatment time. Prior to implementing the OMT protocol, patients underwent mapping and then were readmitted for Y90 administration following dose procurement. This resulted in unnecessary delays in treatment administration or inability to deliver the dose due to disease progression or worsening general condition. The study included patients who were diagnosed with HCC and discussed in HCC multidisciplinary meetings where Y90 treatment was decided. The study excluded patients who underwent mapping only without proceeding to Y90 infusion due to high pulmonary shunt fraction or improper vascular anatomy that preclude safe treatment administration. Patients with lost to follow-up or lack of follow-up imaging after treatment were excluded.

The study was approved by the institutional review board (study number NRR24/046/10, approval number 0000066324) and informed consent was waived due to the retrospective nature of the study.

## 3. Statistical Analysis

Statistical analysis was performed using SPSS version 29. Continuous variables were reported as mean and standard deviation or median and interquartile range. Categorical variables were presented as frequency and percentage. To compare continuous variables among independent groups, either the *t*-test or Mann–Whitney U test was used, depending on the distribution of the data. For categorical variables, the chi-squared test or Fisher’s exact test was used as appropriate. Survival analysis was conducted by Kaplan–Meier and Cox regression. A two-sided *p*-value less than 0.05 was considered statistically significant for all tests. All variables with a *p*-value < 0.2 were included in the final multivariate model. These variables are as follows: age, cirrhosis, splenomegaly, ascites, varices, BCLC stage (CD vs. AB), portal vein thrombosis, AST, alkaline phosphatase, lymphocyte count, creatinine, ALBI score, INR, whole liver volume, tumor volume, liver background dose, lung dose, and Y-90 dose.

## 4. Results

A total of 185 patients (69.2% males) were included in the study, with 88 (47.6%) who underwent TARE before the implementation of the OMT system in 2021 (Group 1) and 97 (52.4%) afterwards (Group 2). The mean age of the entire cohort was 71 years (±12), with no significant difference between the two groups (*p* = 0.807) (Table 1). A significantly larger number of patients treated before 2021 had an ECOG score of 0 (*p* < 0.001). Patients treated after 2021 more often had unilobar involvement, while those treated earlier were more likely to present with multifocal disease (*p* = 0.041). More patients with PVTT3 and PVTT4 were treated after the implementation of the OMT protocol (*p* = 0.009). The target perfused liver volume was smaller in the OMT group 523.7 ± 316.6 cc compared to 860 ± 457.8 cc in the pre-OMT group (*p* = 0.005) without significant differences in the tumor volume or target tumor dose (Table 1).

The median time from multidisciplinary team (MDT) decision to transarterial radioembolization (TARE) was 22 days (IQR 10 to 43) across the entire cohort. When stratified by treatment period, the median time was significantly longer before OMT at 37 days (23 to 57), compared to 15 days (8 to 22) after OMT implementation (*p* < 0.001). The MDT to TARE interval was not associated with improved survival (HR: 1, 95% CI: 0.995 to 1.005, *p* = 0.994).

The median time from mapping to the first TARE procedure was 7.5 days (IQR: 1 to 23) for the overall cohort. Prior to OMT implementation, this interval was significantly longer at 21 days (14 to 33), compared to 1 day (1 to 5) after OMT (*p* < 0.001). However, the mapping to TARE interval was not significantly associated with overall survival (HR: 1.002; 95% CI: 0.999 to 1.005; *p* = 0.160).

The median follow-up time after first TARE treatment was 10.8 months for the entire cohort. The median survival from the first TARE procedure was 1.4 years (95% CI: 1.1 to 1.6) overall. When stratified by treatment period, patients treated before OMT had a median survival of 1.5 years (95% CI: 1.2 to 1.9), while those treated after OMT implementation had a median survival of 1.2 years (95% CI: 0.9 to 1.6). The difference in survival between the two periods was not statistically significant (*p* = 0.415).

**Figure 1:**
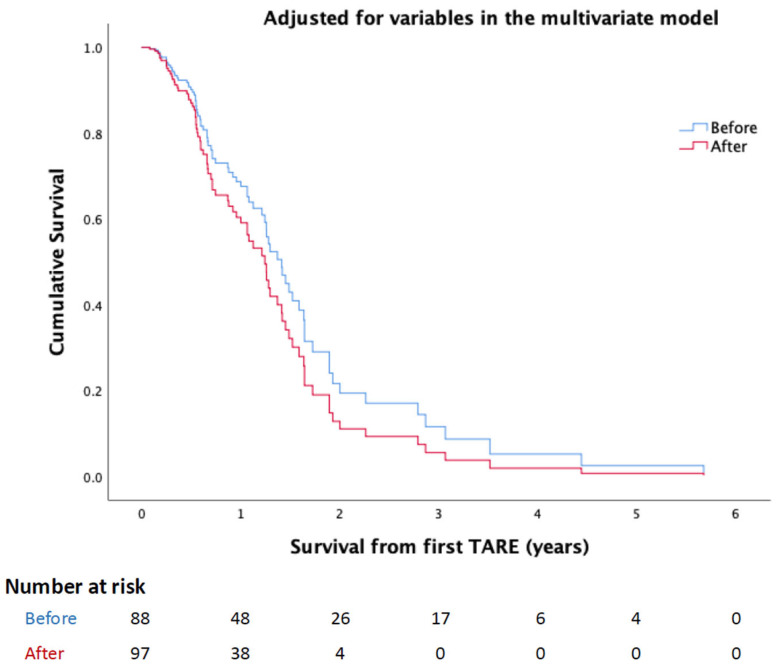
KM curve demonstrates the survival difference before and after implementing the single session OMT protocol after adjusting all variables in the multivariate model.

The survival difference before and after OMT was insignificant even after adjusting for all the variables in the multivariate model (HR: 1.343, 95% CI: 0.75 to 2.406, *p* = 0.321) (Figure 1).

## 5. Discussion

Transarterial radioembolization (TARE) using Yttrium-90 (Y-90) microspheres is a well-established treatment for hepatocellular carcinoma (HCC), particularly in patients who are not candidates for surgical resection or ablation [3,4,16,17]. Traditionally, TARE involves a two-stage process: an initial mapping angiogram with technetium-99m macroaggregated albumin (Tc-99m MAA) simulation followed days to weeks later by the therapeutic delivery of Y-90 microspheres [18,19]. While effective, this bifurcated model introduces logistical challenges, including multiple hospital admissions, increased patient anxiety, and delays in treatment delivery.

Several measures have been recently introduced to optimize and streamline the workflow of Y90 treatments including a transradial approach, Order–Map–Treat (OMT) and Flexdose programs, single day/admission treatments, single session Y90 treatment without prior mapping, and personalized dosimetry software [15,20,21,22,23]. Such compressed timelines and workflow optimizations are especially valuable when rapid treatment decisions are clinically indicated, such as in patients at risk of tumor progression or portal vein thrombosis [24,25].

Recent studies have advocated for a single day or single admission model, often referred to as Order–Map–Treat (OMT), which consolidates pre-treatment mapping, dosimetry, and therapy into a single hospital visit [24,25]. This approach has demonstrated feasibility, safety, and improved efficiency in patient care pathways [24,26,27]. The OMT workflow is particularly relevant in the context of streamlined interventional oncology programs. Prior studies reported their experience of delivering both components of the TARE procedure on the same day during the COVID-19 pandemic to maintain the service and minimize hospital stays [22,23,24,25,27,28,29]. These studies confirmed the feasibility and safety of the same day protocol, offering a new approach to mitigate the logistical challenges and potential health risks associated with the conventional protocol of two separate admissions. Li et al. reported a study of 34 TARE procedures with resin microspheres in 26 patients who underwent same day treatment including the pretreatment mapping and lung shunt fraction study. All procedures were technically successful and only 18% of patients were admitted briefly for observation and symptom management with a mean total procedure time of 4.2 h [27].

The introduction of the Flexdose delivery program allows for the personalization of the administered dose based on the pretreatment angiography and intraprocedural cone-beam CT and offers the possibility of splitting 90Y resin microsphere activity as needed. This feature of the resin microspheres provides flexibility for changes in the treatment plan and allows for the customization of the dose and number of particles based on the tumor characteristics including size, vascularity, and number of supplying vessels [24].

Our institution has initially adopted the same day protocol in which both the mapping and the infusion are performed in one day. However, this was faced by several logistic challenges including the need to maintain the arterial access and catheter position for a longer time, time management and organization between the IR and nuclear medicine departments, and patient discomfort due the prolonged procedural and imaging times. Therefore, we modified the protocol to perform the mapping on the first day and the infusion on the following day. This offers more flexibility in time management and allows for better planning and dose calculation and reduces patient discomfort. Although this program has not shown significant survival benefits compared to the conventional treatment protocol, the reduction in time to treatment was evident. This is concordant with a prior study that shows significant reduction in time from the IR clinic visit to treatment from 61 days in the conventional groups compared to 26.5 days in the single session treatment group (*p* < 0.001) [23]. Nevertheless, the successful implementation of OMT requires robust pre-procedural imaging, patient selection, and coordination between nuclear medicine, interventional radiology, and radiation safety teams. Use of cone-beam CT, same-day SPECT/CT, and real-time dosimetry software has enabled the accurate planning and confident administration of therapeutic doses within a single hospital visit.

Another iteration of TARE protocols is the administration of Y90 treatment without prior lung shunt fraction or SPECT/CT study in select patients. This approach utilizes intra-procedural imaging—primarily cone-beam CT—to assess the hepatic arterial anatomy and exclude any extrahepatic gastrointestinal enhancement, thereby eliminating the need for a separate mapping procedure. In a recent case series, Berman et al. demonstrated that mapping-free single-session ablative Y-90 treatment was technically successful in 88% of patients, with no reported cases of radiation pneumonitis or non-target embolization [30]. Gabr et al. proposed streamlining radioembolization in UNOS T1/T2 hepatocellular carcinoma by eliminating lung shunt estimation. Patients with solitary HCC or ≤3 tumors ≤ 3 cm with no prior history of transjugular intrahepatic portosystemic shunt (TIPS) can safely receive selective TARE without MAA study with no radiation risk to the lungs [28]. A similar finding was observed by Mohnasky et al., who compared a single session Y90 resin radiation segmentectomy without MAA to conventional Y90 glass protocol in select organ procurement and transplantation network (OPTN) stage T2 HCC patients without macrovascular tumor invasion (MVI) or TIPS. [23] There were no ≥grade 3 adverse events and complete response was achieved in 11/12 of the single session patients [23]. Eliminating the mapping session may be associated with cost reduction from nearly USD 64,000 to USD 38,000 [23]. Kim et al. retrospectively compared 100 consecutive patients with HCC within the Milan criteria who underwent regular TARE (n = 38) to streamlined TARE without mapping (n = 62). There was no difference in serious adverse events, complete response, or time-to-progression between the treatment protocols, and they concluded that eliminating mapping was effective and safe in HCC patients within Milan criteria [22]. In a healthcare resource group-based analysis of costs from a national payer, it was estimated that 856 patients per annum would be eligible for SIRT treatment for unresectable HCC in England. OMT would be anticipated to save GBP 2842 per patient versus performing SIRT without OMT. OMT reduces the number of hospital visits required for SIRT by 50%, resulting in financial savings from the Department of Health and Social Care’s perspective, time savings from the patient perspective, and reduced CO2 emissions arising from patient transport [31].

This study is limited by its retrospective nature, relatively small sample size, and some differences in the baseline characteristics between the treatment groups. Patients in the pre-OMT group were healthier with better ECOG status and more multinodular disease. On the other hand, patients in the OMT group had more unilobar disease and greater percentage of more advanced PVTT. These differences may be related to selection bias and changes in experience with TARE treatments, which may ultimately translate into differences in survival outcomes. The lack of survival gain could be attributed to these differences in baseline characteristics prior to and after implementing the single admission OMT protocol.

## 6. Conclusions

The single session OMT approach to Y-90 TARE represents a paradigm shift toward more patient-centered, efficient, and potentially cost-effective care for HCC. As centers gain experience and refine protocols, same-day admission TARE, or even TARE without mapping may become standard practice in appropriately selected patients.

## Figures and Tables

**Table 1:** Comparative baseline characteristics of patients treated with TARE before and after 2021.

	Total	<2021	≥2021	*p*-Value
N	185	88 (47.6%)	97 (52.4%)	
Age (years)	71 ± 12	71 ± 12	71 ± 12	0.807
Gender				
Female	57 (30.8%)	25 (28.4%)	32 (33%)	0.527
Male	128 (69.2%)	63 (71.6%)	65 (67%)	
Body Mass Index	29 ± 8	30 ± 9	28 ± 7	0.682
ECOG Performance Status				
0	114 (62.3%)	69 (80.2%)	45 (46.4%)	<0.001
1	51 (27.9%)	13 (15.1%)	38 (39.2%)	
2	12 (6.6%)	3 (3.5%)	9 (9.3%)	
3	6 (3.3%)	1 (1.2%)	5 (5.2%)	
Diabetes Mellitus	130 (70.3%)	66 (75%)	64 (66%)	0.200
Prior interventions				
None	118 (63.8%)	55 (62.5%)	63 (64.9%)	0.011
Ablation	6 (3.2%)	0 (0%)	6 (6.2%)	
Surgery	2 (1.1%)	0 (0%)	2 (2.1%)	
Systemic	5 (2.7%)	1 (1.1%)	4 (4.1%)	
Transarterial Chemoembolization/ Transarterial Embolization	52 (28.1%)	30 (34.1%)	22 (22.7%)	
TARE	2 (1.1%)	2 (2.3%)	0 (0%)	
Cirrhosis	151 (81.6%)	73 (83%)	78 (80.4%)	0.707
Child score				
A5	52 (28.1%)	18 (20.5%)	34 (35.1%)	0.218
A6	79 (42.7%)	44 (50%)	35 (36.1%)	
B7	32 (17.3%)	16 (18.2%)	16 (16.5%)	
B8	10 (5.4%)	4 (4.5%)	6 (6.2%)	
B9	3 (1.6%)	1 (1.1%)	2 (2.1%)	
C10	1 (0.5%)	0 (0%)	1 (1%)	
Etiology of cirrhosis				
Cryptogenic	84 (45.4%)	40 (45.5%)	44 (45.4%)	0.029
Hemochromatosis	1 (0.5%)	1 (1.1%)	0 (0%)	
Hep B	34 (18.4%)	16 (18.2%)	18 (18.6%)	
Hep C	50 (27%)	28 (31.8%)	22 (22.7%)	
Nonalcoholic fatty liver disease	15 (8.1%)	2 (2.3%)	13 (13.4%)	
Schistosomiasis	1 (0.5%)	1 (1.1%)	0 (0%)	
BCLC stage				
0	20 (10.8%)	15 (17%)	5 (5.2%)	0.091
A	56 (30.3%)	23 (26.1%)	33 (34%)	
B	64 (34.6%)	31 (35.2%)	33 (34%)	
C	42 (22.7%)	18 (20.5%)	24 (24.7%)	
D	3 (1.6%)	1 (1.1%)	2 (2.1%)	
Extent of intrahepatic disease				
Bilobar	28 (15.5%)	13 (15.5%)	15 (15.5%)	0.041
Multifocal	55 (30.4%)	33 (39.3%)	22 (22.7%)	
Segmental	43 (23.8%)	20 (23.8%)	23 (23.7%)	
Unilobar	55 (30.4%)	18 (21.4%)	37 (38.1%)	
Replacement of liver by tumor				
Less than 25%	128 (70.3%)	55 (63.2%)	73 (76.8%)	0.184
25–50%	36 (19.8%)	21 (24.1%)	15 (15.8%)	
50–75%	16 (8.8%)	10 (11.5%)	6 (6.3%)	
>75%	2 (1.1%)	1 (1.1%)	1 (1.1%)	
Largest lesion size (cm)	5.8 ± 4	6.3 ± 4	5.5 ± 3.9	0.181
Portal Vein Tumoral Thrombosis	55 (30.6%)	26 (30.6%)	29 (30.5%)	
PVTT 1	6 (3.2%)	4 (4.5%)	2 (2.1%)	0.009
PVTT 2	24 (13%)	16 (18.2%)	8 (8.2%)	
PVTT 3	16 (8.6%)	3 (3.4%)	13 (13.4%)	
PVTT 4	8 (4.3%)	2 (2.3%)	6 (6.2%)	
Splenomegaly	64 (34.6%)	28 (31.8%)	36 (37.1%)	0.536
Ascites	36 (19.5%)	16 (18.2%)	20 (20.6%)	0.713
Varices	40 (21.6%)	16 (18.2%)	24 (24.7%)	0.290
Whole liver volume	1653.2 ± 497	1654.2 ± 487.3	1652.4 ± 507.5	0.981
Target perfused liver volume	638.2 ± 399.6	860 ± 457.8	523.7 ± 316.6	0.005
Tumor volume	283.2 ± 399.1	284.7 ± 321.5	282 ± 454.9	0.965
Y90 Dose	1.6 ± 0.8	1.7 ± 0.7	1.4 ± 0.8	0.006
Target tumor dose	141.2 ± 58	146.5 ± 47.9	137.4 ± 64.2	0.333
Treated lobe				
Left	31 (16.8%)	11 (12.5%)	20 (20.6%)	0.165
Rad segmentectomy	12 (6.5%)	3 (3.4%)	9 (9.3%)	
Right	130 (70.3%)	63 (71.6%)	67 (69.1%)	

## Data Availability

The data presented in this study are available on request from the corresponding author.
